# Pre-treatment tumour PET metrics and clinical outcomes of anal cancer in patients living with and without HIV

**DOI:** 10.2340/1651-226X.2025.40680

**Published:** 2025-04-24

**Authors:** Michael Pennock, N. Patrik Brodin, Christian Velten, Megi Gjini, Nitin Ohri, Chandan Guha, Shalom Kalnicki, Wolfgang A. Tomé, Madhur K. Garg, Rafi Kabarriti

**Affiliations:** Department of Radiation Oncology, Montefiore Einstein Comprehensive Cancer Center, Albert Einstein College of Medicine, Montefiore Medical Center, Bronx, NY, USA

**Keywords:** Anus neoplasms, HIV, positron-emission tomography, prognostic factors, metrics

## Abstract

**Background/purpose:**

To investigate if pre-treatment tumour positron-emission tomography (PET) metrics’ prognostic efficacy changes with HIV or viral load (VL) in anal squamous cell carcinoma (ASCC).

**Materials and methods:**

Consecutive patients treated with definitive radiation therapy (RT) for non-metastatic ASCC from 2005 to 2021 at one institution were retrospectively identified. Patient demographic and clinical data, including HIV status and pre-treatment VL, were tabulated. Pre-treatment PET metrics were calculated with semi-automatic gradient-based segmentation algorithms. Cox-proportional-hazard and Kaplan-Meier modelling were used to investigate tumour PET metrics and outcomes: overall survival (OS), progression-free survival (PFS), and locoregional control (LRC).

**Results:**

A total of 175 patients were included: 110 HIV-negative and 65 patients living with HIV (PLWH). Nineteen PLWH had detectable pre-treatment VL. Median follow-up was 58 months (interquartile range [IQR]: 28–99), with 28 locoregional failures and 31 deaths. Five-year LRC, PFS, and OS was 84%, 73%, and 86%, respectively. There was no significant difference in LRC, PFS, or OS between HIV-negative patients and PLWH. 156 patients had available pre-treatment PET scans. Metabolic tumour volume and total lesion glycolysis were significantly associated with LRC and PFS on multivariate Cox analysis for the entire cohort (*p* ≤ 0.02), and HIV-negative patients on Cox sub-group analysis (*p ≤* 0.01). No association between PET metrics and outcomes was seen for PLWH.

**Interpretation:**

Outcomes were comparable between HIV-negative patients and PLWH. Pre-treatment PET metrics were validated as significantly predicting outcomes for the entire cohort and HIV-negative patients, not PLWH. This may be from small numbers of PLWH patients, or non-specific uptake in patients with uncontrolled HIV reducing PET’s prognostic efficacy.

## Introduction

Anal squamous cell carcinoma (ASCC) is an uncommon malignancy, with approximately 8300 new diagnoses and 1280 deaths per year in the United States [1]. HIV infection and lower CD4 levels are risk factors [2]. The primary treatment for non-metastatic ASCC involves radiation therapy (RT) and concurrent chemotherapy (CHT, CRT), consisting of 5-fluorouracil (5-FU) and mitomycin-C (MMC), and surgery for salvage [3]. CRT is standard of care [4]. Modern RT techniques like intensity-modulated RT (IMRT) provide improved outcomes and less toxicity [5]. Prior studies have assessed ASCC characteristics individually associated with locoregional failure (LRF) after CRT, and poor prognostic features include stage III, HIV infection, male sex, large tumours (T3+), HPV, or node-positive (N+) disease [6, 7].

Positron-emission tomography (PET) with computed tomography (CT, PET/CT) allows for improved staging, target delineation, and sensitivity over PET or CT alone [8]. Studies assessing PET/CT and incorporated metrics have led to its recommendation for pre-treatment staging because metrics such as pre-treatment metabolic tumour volume (MTV) burden and maximum standardized-uptake value (SUVmax) predict recurrence and survival, while total lesion glycolysis (TLG), which incorporates volume and PET-avidity to estimate tumour burden, is thought to be predictive but has not been fully explored [9–11]. While clinical predictors such as HPV and N3 stage have historically demonstrated predictive efficacy, prognosis and outcomes after CRT can be improved with pre-treatment primary-tumour PET metrics such as MTV, TLG, or Z-normalized combination of MTV and SUV_peak_ (ZMP) [7].

Per sensitivity of 86% (which can differ widely), specificity of 79%, wide heterogeneity in PET-detection and reference standards, PET deficits in resolution of small targets, and high false-positive rates in the setting of infections causing lymphadenopathy, verified nodal PET metrics have yet to be explored or developed [11].

ASCC rates increased five-fold since the introduction of highly-active antiretroviral therapy (HAART), and patients with long-term HIV had higher ASCC rates than those with HIV <5 years [12]. HAART duration does not reduce ASCC risk [12]. ASCC incidence is rising in HIV-infected patients living longer on HAART [12].

HIV infection induces lymphocyte glycolysis and PET-activation patterns indicative of viral load (VL) and HIV disease-state [13, 14]. In immunocompromised patients, anogenital neoplasms are more aggressive [15]. Attempting to ascertain prognosis in ASCC patients living with HIV (PLWH) by PET metrics may be complicated by infection, VL, and HAART adherence, which is largely unexplored [16]. There is conflicting data, with some studies showing PLWH and ASCC have worse outcomes than HIV-negative ASCC patients [17]. Other studies have shown CRT is well-tolerated by PLWH and outcomes are similar to those for HIV-negative patients [18].

Exploration is needed into the knowledge gap for how clinical outcomes and PET metrics’ predictive efficacy in ASCC patients changes with HIV and VL. HIV’s effect on ASCC outcomes is under-studied [10]. This novel study investigates and validates the association between pre-treatment tumour PET metrics and clinical outcomes in a large cohort of ASCC patients inclusive of many PLWH and varying VL, treated with definitive RT. The study explores MTV, SUVmax, and TLG as pre-treatment PET metrics [9, 10], which may differentially predict clinical outcomes in ASCC patients from HIV and VL.

## Materials and methods

### Design, setting, and data source

In this institutional review board (IRB)-approved retrospective cohort study, all patients at a single institution who underwent definitive RT for ASCC were retrospectively identified from clinical and treatment records between 2005 and 2021. Informed consent was not required per the retrospective nature and minimal risk.

### Participants

Patients underwent staging PET/CT with [18F]fluorodeoxyglucose (FDG) tracer as part of routine clinical practice, pathologic confirmation, and curative-intent RT commonly prescribed by their physician to 50.4–54 Gy in 1.8-Gy fractions using IMRT for non-metastatic ASCC, stages I-III. Patients with surgically-excised small tumours were excluded. Chemotherapy was 5-FU and MMC, capecitabine and MMC, cisplatin, or cetuximab. MMC was prescribed as two 10 mg/m^2^ infusions as one cycle on days 1 and 29 of RT.

### Outcomes

All patients were analysed for survival, recurrence, or progression. Loco-regional control (LRC) was time from last RT treatment to locally- or regionally-recurrent disease based on pathologic sampling or imaging. Progression free survival (PFS) was time from last RT treatment to local, locoregional, or distant recurrence of disease or death. Overall survival (OS) was time elapsed from date of last RT treatment to date of death or censored at last follow-up.

### Variables and clinical predictors

Clinical and demographic data to most recent follow-up, age at diagnosis, RT dose and fractionation, chemotherapy, gender, HIV status, HPV status, most recent pre-treatment VL, smoking status, stage, comorbidities, Charlson Comorbidity Index, and death date and cause were tabulated. Socioeconomic status (SES) was calculated by a representative summary score based on home address, neighbourhood information considering median household income, median value of housing units, occupation and education of inhabitants, and percentage of households receiving interest or net rental income.

Tumor, lymph node, and metastasis (TNM) stage and tumour diameter were determined at multidisciplinary oncologic conferences based on available diagnostic information, including clinical assessment, anoscopy, MRI, and PET/CT. Contours were drawn in Eclipse planning software (Varian Medical Systems, Palo Alto, CA).

### PET/CT metric predictors

PET scanning was performed on a Siemens Biograph mCT (Siemens Medical Solutions, Erlangen, Germany) with 3D point-spread function, time-of-flight reconstruction, and a 400 × 400 reconstruction matrix (2-mm plane resolution, 3-mm slice spacing). The patients fasted for 6 h prior to FDG injection, and images were obtained 1-h post-injection. All patients included had acceptable blood glucose levels below the upper threshold of 7 mmol l^−1^.

Pre-treatment PET/CT was fused with CT simulation in ARIA/Eclipse^®^ treatment planning software (Varian Medical Systems, Palo Alto, CA) and used to delineate target volumes. On treatment, patients were seen by a radiation oncologist weekly for toxicity assessment. Patients were seen for follow-up in clinic per National Comprehensive Cancer Network (NCCN) guidelines for toxicity, disease status, management, or surveillance. Last date of follow-up was extrapolated from the chart, as were locations and dates for recurrence, distant progression, and treatment breaks.

Pre-treatment PET/CT’s were imported to MIM^®^ (MIM Software, Cleveland OH) for a semiautomatic, gradient-based, segmentation-tool algorithm (PET_Edge) to define the primary tumour and calculate primary-tumour PET metrics, including SUVmax, MTV, and TLG. PET_Edge was used only for the contoured primary gross-tumour volume (GTV) within the anal canal (Supplementary Figure 1). SUVmax was the GTV voxel with the highest SUV avidity, correcting for patient weight and injected activity. TLG was MTV (designating volume) multiplied by SUVmean (designating avidity) of GTV. Algorithm- and adaptive methods, such as PET Edge, are gradient-based segmentation methods that define metabolic tumour boundary via SUV gradient between tumour and surrounding tissues.

### Statistical analysis

Variables included gender, ethnicity, race, stage, chemotherapy, local recurrence, regional recurrence, distant recurrence, death, age, SES, Charlson Comorbidity Index, treatment duration (first to last day of RT), follow-up duration, and PET metrics.

Patients without pre-treatment PET/CT scans were excluded from PET-metrics analysis but were included in descriptive statistics. Univariable Cox sub-group models evaluated the individual effect of MTV, TLG, and SUVmax on LRC, PFS, OS in all patients, HIV-negative patients, PLWH with VL, and PLWH without VL. Separate univariable Cox analyses assessed effect of age, HIV status, sex, race, ethnicity, Charlson Comorbidity Index, stage, chemotherapy, smoking, MTV, TLG, and SUVmax on LRC, PFS, and OS. Multivariable Cox proportional hazard models then assessed which risk factors retained their statistical significance for predicting LRC, OS, and PFS.

In Cox proportional-hazard analyses, PET metrics were analysed as continuous variables to allow comparison between groups and to other studies investigating the prognostic value of tumour PET metrics in similar populations. Univariable Cox proportional-hazard modelling was used to generate hazard ratios (HR) and confidence intervals (CI), with two-sided *p* < 0.05 considered statistically significant to exclude non-significant metrics without single impact on outcomes, as well as include significant factors for multivariate analysis.

Separate Kaplan-Meier curves with log-rank comparison of groups assessed the impact of MTV, TLG, SUVmax, and HIV status on OS, PFS, and LRC.

Analysis with *t*-tests and linear regression were performed using Matlab^^®^^ (The Mathworks, Natick, MA, USA) and Stata^®^ (StataCorp, College Station, TX, USA).

## Results

A total of 175 ASCC patients were identified: 110 HIV-negative, 46 PLWH with undetectable VL, and 19 PLWH with VL. Median follow-up time was 52.7 months (IQR:2.0–199.1) for HIV-negative patients and 59.0 months (1.1–185.2) for PLWH. All patients were p16+, thus HPV status was not analysed. All pre-treatment VL’s were within 2 months of day 1 of RT. There were 110 HIV-negative patients. PLWH (median age: 51 years [IQR:32–78]) were significantly younger than HIV-negative patients (median age: 64 years [IQR:39–92]) (*p* < 0.01).

The proportion of women was significantly higher in HIV-negative patients (78%) than in PLWH (43%) (*p* < 0.01) (Table 1).

**Table 1:** Patient and treatment characteristics.

	HIV negative (*n* = 110)	HIV positive (*n* = 65)	*p*
Age (y), median (range)	64 (39, 92)	51 (32, 78)	**< 0.001**
Gender, *n* (%)			
Male	24 (22)	37 (57)	**< 0.001**
Female	86 (78)	28 (43)	
Race and ethnicity, *n* (%)			
Hispanic	51 (46)	30 (46)	**0.024**
Non-Hispanic black	25 (23)	26 (40)	
Non-Hispanic white	20 (18)	4 (6)	
Other/Unknown	14 (13)	5 (8)	
Stage, *n* (%)			
I	11 (10)	12 (19)	**0.021**
II	41 (37)	12 (18)	
III	58 (53)	41 (63)	
Active viral load (VL), *n* (%)			
No	N/A	44 (68)	N/A
Yes		21 (32)	
Charlson comorbidity index, median (range)	4 (2, 13)	9 (7, 17)	**< 0.001**
Chemotherapy, *n* (%)			
None (RT alone)	5 (4)	4 (6)	0.95
5-Fluorouracil (5FU) + Mitomycin	67 (61)	39 (60)	
Xeloda + Mitomycin	36 (33)	21 (32)	
Other/unknown	2 (2)	1 (2)	
Primary tumour SUVmax, median (IQR)	10.9 (2.2–36.1)	8.7 (2.3–26.6)	0.2
Primary tumour MTV (cm^3^), median (IQR)	21.6 (2.6–525.4)	31.8 (3.9–297.0)	0.069
Primary tumour TLG, median (IQR)	101.5 (5.0–2.416)	101.6 (10.2–3.139)	0.59
Socio-economic status	-2.4	-4.3	**0.01**
Follow-up duration, months (range)	52.7 (2.0–199.1)	59.0 (1.1–185.2)	0.88
Treatment duration, days (IQR)	46 (42–52)	47 (43–56)	0.24

MTV: metabolic tumour volume; TLG: total lesion glycolysis. A two-sided *p* < 0.05 is considered statistically significant, marked in bold.

Distribution of race was significantly different between groups, with greater percentages of black PLWH and white or other HIV-negative patients (*p* = 0.024) (Table 1). SES was significantly lower in PLWH (*p* = 0.01) (Table 1). Baseline patient characteristics are shown in Table 1.

A total of 156 patients had available pre-treatment PET/CT scans; 97 HIV-negative and 59 PLWH, including all 19 PLWH with detectable VL. RT was completed for 169 patients: 110 patients received 54 Gy, and 59 patients received 50.4 Gy. There were 5 HIV-negative patients and 4 PLWH that underwent RT alone. Chemotherapy was 5-FU and MMC (67 HIV-negative/39 PLWH), capecitabine and MMC (36 HIV-negative/21 PLWH), or other (2 HIV-negative/1 PLWH). Patients receiving MMC completed two infusions concurrent with RT, except three PLWH who received one infusion. There was no significant difference in treatment duration between HIV-negative patients and PLWH (*p* = 0.24) (Table 1).

Six patients (2 HIV-negative (16 Gy, 40 Gy)/4 PLWH (29 Gy, 34 Gy, 36 Gy, 41 Gy)) did not complete treatment per non-compliance. One of these non-compliant PLWH did not have a pre-treatment PET/CT. Otherwise, they were included in all appropriate analyses. The proportion of stage I (10%), stage II (37%), and stage III (53%) in HIV-negative patients was significantly different from PLWH (19% Stage I, 18% stage II, 63% stage III) (*p* = 0.02) (Table 1).

There were 34 locoregional failures, 31 progressions, and 34 deaths: 9 from ASCC, 4 from other cancers, 12 from non-oncologic causes, and 9 from unknown causes (Table 2). Overall, 3- and 5-year LRC was 89% and 84%; 3- and 5-year PFS was 81% and 73.0%; and 3- and 5-year OS was 90% and 86%.

**Table 2:** Univariate cox sub-group analyses.

	Hazard ratio (95% CI)	*p*
**Locoregional Control (*n* = 156) Hazard ratio (95% CI)**		
HIV-patients (*n* = 97)	loco-regional failures: 18	
MTV (per 10 cm^3^ increase)	1.044 (95% CI: 1.014, 1.075)	**0.004**
SUVmax (per 1 unit increase)	1.067 (95% CI: 0.993, 1.148)	0.078
TLG (per 100 cm^3^ increase)	1.168 (95% CI: 1.082, 1.262)	**<0.001**
PLWH (*n* = 59)	loco-regional failures: 8	
MTV (per 10 cm^3^ increase)	1.110 (95% CI: 1.033, 1.193)	**0.004**
SUVmax (per 1 unit increase)	1.048 (95% CI: 0.939, 1.170)	0.4
TLG (per 100 cm^3^ increase)	1.198 (95% CI: 1.069, 1.341)	**0.002**
PLWH VL (*n* = 19)	loco-regional failures: 3	
MTV (per 10 cm^3^ increase)	0.968 (95% CI: 0.805, 1.165)	0.73
SUVmax (per 1 unit increase)	0.996 (95% CI: 0.738, 1.342)	0.98
TLG (per 100 cm^3^ increase)	0.926 (95% CI: 0.614, 1.397)	0.71
PLWH undetectable VL (*n* = 40)	loco-regional failures: 5	
MTV (per 10 cm^3^ increase)	1.160 (95% CI: 1.058, 1.273)	**0.002**
SUVmax (per 1 unit increase)	1.063 (95% CI: 0.940, 1.202)	0.33
TLG (per 100 cm^3^ increase)	1.258 (95% CI: 1.101, 1.438)	**0.001**
**Progression-Free Survival (*n* = 156) Hazard ratio (95% CI)**		***p***
HIV-patients (*n* = 97)	died or progressed: 35	
MTV (per 10 cm^3^ increase)	1.036 (95% CI: 1.012, 1.060)	**0.003**
SUVmax (per 1 unit increase)	1.066 (95% CI: 1.011, 1.123)	**0.017**
TLG (per 100 cm^3^ increase)	1.159 (95% CI: 1.094, 1.228)	**<0.001**
PLWH (*n* = 59)	died or progressed: 15	
MTV (per 10 cm^3^ increase)	1.064 (95% CI: 1.001, 1.132)	**0.048**
SUVmax (per 1 unit increase)	1.006 (95% CI: 0.922, 1.097)	0.9
TLG (per 100 cm^3^ increase)	1.122 (95% CI: 1.012, 1.245)	**0.029**
PLWH VL (*n* = 19)	died or progressed: 6	
MTV (per 10 cm^3^ increase)	0.965 (95% CI: 0.839, 1.109)	0.61
SUVmax (per 1 unit increase)	0.940 (95% CI: 0.755, 1.169)	0.58
TLG (per 100 cm^3^ increase)	0.898 (95% CI: 0.640, 1.259)	0.53
PLWH undetectable VL (*n* = 40)	Nr of pts died or progressed: 9	
MTV (per 10 cm^3^ increase)	1.094 (95% CI: 1.022, 1.172)	**0.010**
SUVmax (per 1 unit increase)	1.023 (95% CI: 0.926, 1.130)	0.66
TLG (per 100 cm^3^ increase)	1.166 (95% CI: 1.046, 1.300)	**0.006**
**Overall Survival (*n* = 156) Hazard ratio (95% CI)**		***p***
HIV-patients (*n* = 97)	Nr of pts died: 16	
MTV (per 10 cm^3^ increase)	1.019 (95% CI: 0.974, 1.066)	0.41
SUVmax (per 1 unit increase)	1.094 (95% CI: 1.022, 1.171)	**0.01**
TLG (per 100 cm^3^ increase)	1.125 (95% CI: 1.028, 1.231)	**0.011**
PLWH (*n* = 59)	Nr of pts died: 9	
MTV (per 10 cm^3^ increase)	1.075 (95% CI: 0.993, 1.165)	0.075
SUVmax (per 1 unit increase)	1.018 (95% CI: 0.915, 1.133)	0.74
TLG (per 100 cm^3^ increase)	1.149 (95% CI: 1.010, 1.308)	**0.035**
PLWH VL (*n* = 19)	Nr of pts died: 3	
MTV (per 10 cm^3^ increase)	1.017 (95% CI: 0.863, 1.199)	0.84
SUVmax (per 1 unit increase)	0.996 (95% CI: 0.747, 1.329)	0.98
TLG (per 100 cm^3^ increase)	1.006 (95% CI: 0.700, 1.445)	0.98
PLWH undetectable VL (*n* = 40)	Nr of pts died: 6	
MTV (per 10 cm^3^ increase)	1.091 (95% CI: 0.997, 1.193)	0.057
SUVmax (per 1 unit increase)	1.025 (95% CI: 0.909, 1.155)	0.69
TLG (per 100 cm^3^ increase)	1.167 (95% CI: 1.020, 1.336)	**0.025**

MTV: metabolic tumour volume; TLG: total lesion glycolysis; CI: confidence intervals. A two-sided *p* < 0.05 is considered statistically significant, marked in bold.

At 5 years post-treatment, there were 69 events in HIV-negative patients, 20 events in PLWH with undetectable VL, and 12 events in PLWH with detectable VL (Table 2).

**Figure 1:**
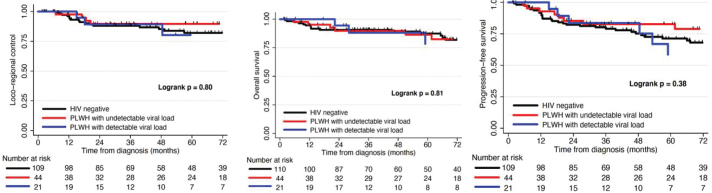
Kaplan Meier curves depicting rates of locoregional control (LRC), progression-free survival (PFS), and overall survival (OS) after definitive therapy for HIV-patients, patients living with HIV (PLWH) without viral load (VL), and PLWH with VL.

There was no significant difference in 3- and 5-year PFS (logrank *p* = 0.38), LRC (logrank *p* = 0.80), or OS (logrank *p* = 0.80) on Kaplan-Meier analysis or Cox analysis between HIV-negative patients, PLWH without VL, and PLWH with VL (Figure 1; Table 3).

**Table 3:** Univariate cox models (n=156).

	Loco-regional control, Hazard ratio (95% CI)	*p*	Progression-free survival, Hazard ratio (95% CI)	*p*	Overall survival, Hazard ratio (95% CI)	*p*
Age	0.99 (0.96, 1.02)	0.62	1.02 (0.99, 1.04)	0.066	1.02 (0.98, 1.05)	0.32
HIV status (positive vs. negative)	0.72 (0.31, 1.65)	0.44	0.68 (0.37, 1.25)	0.22	0.88 (0.39, 1.99)	0.75
Sex (female vs. male)	1.37 (0.58, 3.27)	0.48	0.99 (0.55, 1.78)	0.97	0.91 (0.40, 2.06)	0.82
Race and ethnicity						
Hispanic	(ref)		(ref)		(ref)	
Non-Hispanic black	0.86 (0.34, 2.20)	0.76	0.52 (0.24, 1.11)	0.091	0.60 (0.21, 1.72)	0.34
Non-Hispanic white	1.17 (0.38, 3.63)	0.79	1.29 (0.60, 2.77)	0.52	1.54 (0.54, 4.39)	0.42
Other/declined	1.39 (0.39, 4.96)	0.61	1.61 (0.69, 3.76)	0.27	1.54 (0.43, 5.55)	0.51
Charlson Comorbidity Index (continuous)	1.20 (1.08, 1.34)	**0.001**	1.18 (1.09, 1.27)	**<0.001**	1.20 (1.07, 1.33)	**0.001**
Stage						
I	(ref)		(ref)		(ref)	
II	1.29 (0.25, 6.63)	0.76	1.36 (0.48, 3.81)	0.56	1.38 (0.37, 5.20)	0.64
III/IV	2.38 (0.55, 10.2)	0.25	1.72 (0.67, 4.43)	0.26	1.25 (0.36, 4.39)	0.72
Chemotherapy (Xeloda vs. 5FU)	0.31 (0.09, 1.06)	0.061	1.01 (0.52, 1.98)	0.97	1.23 (0.48, 3.14)	0.66
Smoking					(ref)	
Never	(ref)		(ref)			
Current	0.85 (0.35, 2.09)	0.72	1.18 (0.62, 2.23)	0.62	2.08 (0.80, 5.39)	0.13
Previous	1.28 (0.48, 3.41)	0.63	1.39 (0.67, 2.90)	0.38	2.53 (0.89, 7.23)	0.082
SUVmax (per 1 unit increase)	1.06 (1.00, 1.13)	0.051	1.05 (1.00, 1.10)	**0.041**	1.07 (1.01, 1.13)	**0.028**
MTV (per 10 cm^3^ increase)	1.05 (1.03, 1.08)	**<0.001**	1.04 (1.02, 1.06)	**<0.001**	1.03 (0.99, 1.07)	0.087
TLG (per 100 cm^3^ increase)	1.18 (1.10, 1.25)	**<0.001**	1.15 (1.09, 1.21)	**<0.001**	1.13 (1.05, 1.22)	**0.001**

MTV: metabolic tumour volume; TLG: total lesion glycolysis; CI: confidence intervals. A two-sided *p* < 0.05 is considered statistically significant, marked in bold.

**Figure 2:**
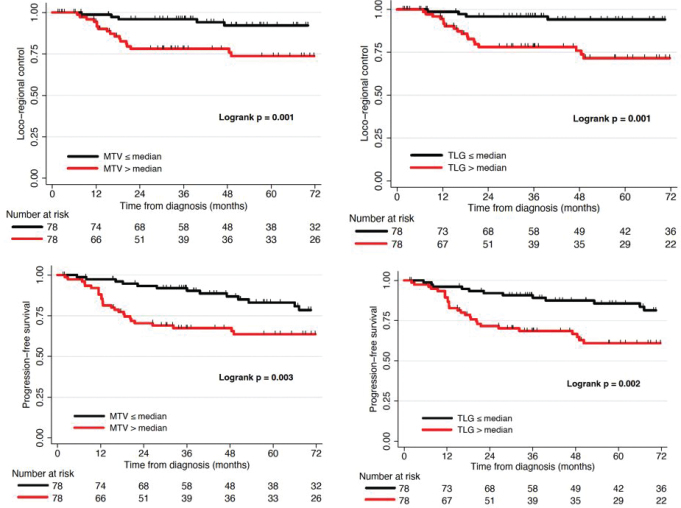
Kaplan Meier curves depicting rates of locoregional control (LRC) and progression-free survival (PFS) after definitive therapy, stratified by metabolic tumour volume (MTV) or total lesion glycolysis (TLG) greater or less than the median.

**Figure 3:**
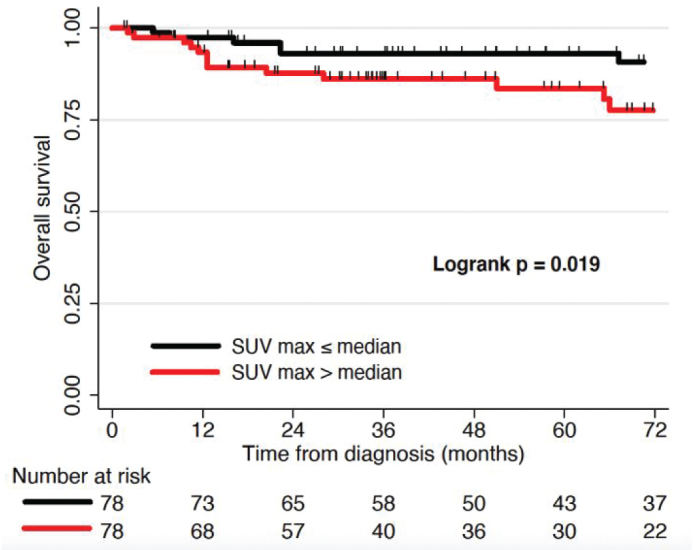
Kaplan Meier curves depicting rates of overall survival (OS) after definitive therapy, stratified by maximum standardized uptake value (SUVmax) greater or less than the median.

For the 156 patients with pre-treatment PET scans, primary tumour MTV and TLG dichotomised at median, 26.5 cm^3^ for MTV (21.6 cm^3^ HIV-negative/31.8 cm^3^ PLWH) and 101.6 cm^3^ for TLG (101.5 cm^3^ HIV-negative/101.6 cm^3^ PLWH) (Table 1), were significantly associated with LRC (*p* = 0.001, *p* = 0.001) and PFS (*p* = 0.003, *p* = 0.002) for all patients on Kaplan-Meier analysis (Figure 2). Primary tumour SUVmax (median 9.4 (10.9 HIV-negative/8.7 PLWH)) was significantly associated with OS on Kaplan-Meier analysis (*p* = 0.02) (Figure 3).

On Cox univariable sub-group analysis of HIV-negative patients, MTV and TLG were significantly associated with LRC (MTV, *p* = 0.004; TLG, *p* < 0.001) and PFS (MTV, *p* = 0.003; TLG, *p* < 0.001), TLG was significantly associated with OS (*p* = 0.011), and SUVmax was significantly associated with PFS (*p* = 0.017) and OS (*p* = 0.010) (Table 2).

MTV and TLG were significantly associated with LRC (MTV, *p* = 0.004; TLG, *p* = 0.002) and PFS (MTV, *p* = 0.048; TLG, *p* = 0.029) for PLWH, and with LRC (MTV, *p* = 0.002; TLG, *p* = 0.001) and PFS (MTV, *p* = 0.010; TLG, *p* = 0.006) for PLWH with undetectable VL. TLG also predicted OS for PLWH (*p* = 0.035) and PLWH with undetectable VL (*p* = 0.025) (Table 2). There was no association between PET metrics and outcomes for PLWH with detectable VL.

Primary tumour TLG was significantly associated with OS (*p* = 0.001), and MTV and TLG were significantly associated with LRC (MTV, *p* < 0.001; TLG, *p* < 0.001) and PFS (MTV, *p* < 0.001; TLG, *p* < 0.001) for all patients on Cox univariable analysis, while SUVmax was significantly associated with PFS (*p* = 0.041) and OS (*p* = 0.028) (Table 3).

Stage was not a significant predictor of outcomes in univariable Cox analyses (Table 3), although there was a significant association between stage and pre-treatment PET metrics. Median MTV was 17.1 cm^3^, 17.8 cm^3^ and 33.0 cm^3^ for patients with stage I, II and III disease, respectively (*p* = 0.008), and the corresponding median TLG was 55.4 cm^3^, 57.3 cm^3^ and 158.9 cm^3^, respectively (*p* = 0.03).

Charlson Comorbidity Index significantly predicted clinical outcomes in all patients on univariable analysis (*p* ≤ 0.001) (Table 3). Locoregional failures occurred in all stages (Table 3).

In multivariable Cox models, after adjusting for age, stage, HIV status, and smoking status, MTV was significantly associated with LRC (*p* = 0.001), PFS (*p* < 0.001), and OS (*p* = 0.019); and TLG was significantly associated with LRC (*p* < 0.001), PFS (*p* < 0.001), and OS (*p* = 0.037) for the entire cohort (Table 4). SUVmax was not a significant predictor in multivariable analysis. LRC, PFS, and OS remained similar for HIV-negative patients and PLWH after multivariable analysis adjustment (Table 4).

**Table 4:** Multivariable cox models.

	Locoregional Control (*n* = 156) Hazard ratio (95% CI)	*p*	Progression-free Survival (*n* = 156) Hazard ratio (95% CI)	*p*	Overall Survival (*n* = 156) Hazard ratio (95% CI)	*p*
**Model with MTV**						
MTV (per 10 cm^3^ increase)	1.05 (1.02, 1.08)	**0.001**	1.04 (1.02, 1.07)	**< 0.001**	1.05 (1.01, 1.09)	**0.019**
HIV (Positive vs. negative)	0.52 (0.18, 1.45)	0.21	0.84 (0.40, 1.78)	0.66	1.10 (0.40, 3.01)	0.86
Age (Continuous)	0.97 (0.93, 1.01)	0.20	1.02 (0.99, 1.06)	0.12	1.03 (0.99, 1.08)	0.17
Stage						
I	(ref)		(ref)		(ref)	
II	2.93 (0.36, 24.0)	0.32	1.99 (0.55, 7.23)	0.29	1.45 (0.30, 7.10)	0.65
III/IV	4.06 (0.64, 25.7)	0.14	2.09 (0.66, 6.60)	0.21	1.13 (0.26, 4.90)	0.87
Chemotherapy						
5FU + Mitomycin	(ref)		(ref)		(ref)	
Xeloda + Mitomycin	0.35 (0.10, 1.22)	0.10	1.09 (0.54, 2.20)	0.81	1.26 (0.47, 3.35)	0.65
None	4.32 (0.65, 28.8)	0.13	2.56 (0.70, 9.34)	0.16	0.80 (0.08, 8.08)	0.85
Smoking						
Never	(ref)		(ref)		(ref)	
Current	1.12 (0.41, 3.07)	0.82	1.85 (0.88, 3.89)	0.10	3.28 (1.10, 9.78)	**0.033**
Previous	1.95 (0.63, 6.01)	0.25	1.85 (0.82, 4.20)	0.14	2.88 (0.89, 9.33)	0.078
**Model with TLG**						
TLG (per 100 cm^3^ increase)	1.20 (1.11, 1.30)	**< 0.001**	1.17 (1.10, 1.24)	**< 0.001**	1.16 (1.08, 1.26)	**< 0.001**
HIV (Positive vs. negative)	0.39 (0.13, 1.18)	0.096	0.76 (0.35, 1.63)	0.48	0.99 (0.35, 2.82)	0.98
Age (Continuous)	0.96 (0.92, 1.01)	0.11	1.02 (0.99, 1.05)	0.17	1.03 (0.99, 1.07)	0.20
Stage						
I	(ref)		(ref)		(ref)	
II	3.59 (0.40, 31.8)	0.25	1.97 (0.53, 7.35)	0.31	1.38 (0.27, 7.03)	0.70
III/IV	3.56 (0.52, 24.3)	0.19	1.68 (0.51, 5.54)	0.39	0.91 (0.20, 4.18)	0.91
Chemotherapy						
5FU + Mitomycin	(ref)		(ref)		(ref)	
Xeloda + Mitomycin	0.41 (0.12, 1.45)	0.17	1.20 (0.59, 2.41)	0.61	1.34 (0.50, 3.59)	0.56
None	7.15 (0.97, 52.9)	0.054	3.20 (0.85, 12.0)	0.085	0.96 (0.09, 9.82)	0.97
Smoking						
Never	(ref)		(ref)		(ref)	
Current	1.16 (0.43, 3.13)	0.76	1.77 (0.86, 3.63)	0.12	3.04 (1.07, 8.64)	**0.037**
Previous	2.22 (0.70, 7.02)	0.17	1.69 (0.75, 3.83)	0.21	2.35 (0.73, 7.57)	0.15

MTV: metabolic tumour volume; TLG: total lesion glycolysis; CI: confidence intervals. Proportional-hazard modelling was used to generate hazard ratios (HR) and confidence intervals (CI), with two-sided p < 0.05 considered statistically significant.

*HIV and Charlson comorbidity Index cannot be used together as they are highly collinear, therefore only HIV was used in the multivariable models.

Charlson Comorbidity Index was not included in multivariable models as it is strongly driven by HIV status. HIV and Charlson Comorbidity Index could not both be included due to high levels of collinearity. MTV and TLG were significant risk factors (Tables 3 and 4) in HIV-negative patients and PLWH, but appeared to not be significant when analysed in PLWH with active VL, although that group had a very limited sample size (*n* = 19).

## Discussion

This study, novel for large ASCC and PLWH populations, demonstrated that patients with ASCC had similar clinical outcomes and minimal toxicity with definitive therapy regardless of HIV status, and should be considered for definitive therapy [19–21]. Pre-treatment PET metrics, especially MTV and TLG, were significantly associated with 3- and 5-year clinical outcomes, and demonstrated significant prognostic utility in the entire cohort. The strongest predictors were PET metrics and Charlson Comorbidity Index. These findings expand upon prior studies supporting the utility of pre-treatment PET/CT in ASCC [7, 10, 22]. Future large-scale studies will verify the prognostic value of mid- or post-treatment PET metrics [23, 24].

This study supports MTV and TLG as holding the most prognostic value in the ASCC pre-treatment setting, being prognostic for PLWH (possibly not in the small group with active VL), and reflecting findings from previous smaller studies [7, 10, 17]. MTV describes metabolically-active tumour volume, which is associated with GTV or primary-tumour size, and higher MTV values portend worse prognosis as a measure of tumour burden [22]. SUVmax, a ratio of GTV maximum voxel-value to radiotracer dose, is sensitive to noise, patient traits, and imaging parameters [25]. TLG (SUVmean × MTV) estimates tumour burden from volume and avidity, and has been associated with treatment outcome [26]. GTV size may differ from tumour size if GTV includes anorectal circumference, gas, or stool, but this study only used primary tumour.

Different threshold- or algorithm-based methods have been proposed. MTV and TLG with a threshold of SUV 2.5 had shown predictive value in prior studies [27]. There was debate whether absolute or relative thresholds are ideal for pre-treatment MTV delineation. Absolute thresholds may generate inaccurate MTV values if tumour uptake is significantly outside threshold, while relative- and gradient-thresholds may distort MTV via tumour size, signal-background ratios, or PET reconstruction [25]. Threshold-based segmentation can cause information loss with heterogenous uptake [25].

Algorithm-based and adaptive methods, such as PET Edge in this study, represent optimised gradient-based segmentation that overcomes prior shortcomings by defining tumour boundary via SUV gradient between tumour and normal tissues [28]. This is optimal for assessing tumour dimensions and MTV because they segment more accurately than fixed thresholds for wide ranges of uptake and size [25, 27]. Future studies can assess thresholding in clinically-relevant PET metrics, as well as further investigate SUVmax, which demonstrated uncertain predictive value. This study contributes to evidence that MTV and TLG best show anogenital tumour burden with accurate segmentation [29], indicate disease aggressiveness in this population, may be more sensitive than standard staging (stage did not significantly predict outcomes here), and may predict treatment-resistant disease, failure risk, and indications for treatment intensification.

Pre-treatment PET metrics predicted clinical outcomes for PLWH without VL, but not for PLWH with VL. This may be due to limited patient numbers in this cohort, making it difficult to assess a prognostic factor’s strength between groups with a large difference in patient number, or VL or immunogenic cancer-HIV interactions reducing PET’s prognostic efficacy. HIV infection can cause PET whole-body and lymphoid uptake [14, 30, 31]. VL may affect thresholding through signal-background ratios and uptake [25, 27]. Future studies can investigate whether this study’s findings for PLWH are from low power, any HIV state necessitating different PET segmentation or thresholding per undiscovered factors in our heterogenous PLWH population, or an interaction between cancer, HIV, and CRT that affects outcomes, analogous to CRT affecting bone-marrow injury and compensatory response [32]. There is potential to further explore whether PET metrics are prognostic in PLWH with active VL with more patients. HIV or HAART can affect immune-cell counts, infection risk, and toxicity [33], and this impact on PET metrics can be explored.

New PET metrics like ZMP may improve prognosis and identify patients amenable to treatment intensification, as ZMP can identify more aggressive areas within a tumour that may represent dose-painting targets [7, 34]. However, the lack of predictive power of SUVmax in this study, and the comparable predictive strength of ZMP and TLG in prior studies [34], indicates ZMP may not offer significant improvements in dose-painting utility over MTV or TLG.

## Strengths and limitations

This study is notable for a uniquely large ASCC population, an unexplored clinical question that holds importance for a growing group of under-studied ASCC patients, a practical and reproducible method of importing planning scans and PET imaging, a facile gradient-based segmentation tool (PET Edge) to calculate prognostic PET metrics [28, 35], findings confirming pre-treatment PET metrics’ prognostic utility for most ASCC patients, long follow-up, consistent treatment at a single institution allowing minimal variability amongst treatment plans, and intriguing findings indicating the importance of more research into PLWH and ASCC. This study offers validation of pre-treatment PET metrics in ASCC, and how this utility varies with HIV status and VL. Larger, multi-institutional, prospective studies are needed to further explore these results.

Despite the large cohort size and inclusion of a sizable HIV+ population, the study was retrospective at a single institution. PLWH were not stratified by HAART or CRT adherence, as some PLWH may have stopped HAART during CRT and developed VL during and after treatment, while PLWH with active VL may have been non-adherent to CRT, experienced CRT breaks, or been non-adherent to follow-up. These possibilities are largely unexplored outside small studies [16, 17, 36]. Greater numbers of PLWH with and without VL are needed. Among patients that experienced recurrence, future studies will explore the impact of longer duration from biopsy to CRT start, RT course extension [36], and HIV status or VL [37]. This study also analysed two data sets: patients (*n* = 175) for clinical outcomes and patients with PET scans (*n* = 156) for PET metrics.

We did not evaluate PET metrics for nodes. There is a growing body of literature addressing pre-treatment primary-tumour PET metrics, but there is no verified nodal PET metric method, due to wide heterogeneity in sensitivity and specificity of nodal PET detection and prognostication, heterogeneity in reference standards and including all PET-avid nodes or nodes that have been verified as ASCC by histopathology, potential deficits in PET resolution for smaller targets, and high false-positivity rate due to coinfection with aetiologies causing inguinal lymphadenopathy [11]. If future studies are to assess nodal PET metrics, we need to develop methodology to assess pathology-verified nodes, all PET-avid nodes, total or individual nodal avidity, nodal location, node number, or developing a composite score [7, 9, 10, 17].

## Interpretation

Patients with ASCC had similar outcomes regardless of HIV status. Advanced pre-treatment PET metrics, especially MTV and TLG, demonstrated prognostic utility in HIV-negative patients and PLWH and undetectable VL with ASCC, but were not associated with clinical outcomes for PLWH with active VL, perhaps due to small numbers of patients or immunogenic factors that remain to be explored.

## Supplementary Material

Pre-treatment tumour PET metrics and clinical outcomes of anal cancer in patients living with and without HIV

## Data Availability

Research data are stored in an institutional repository and will be shared upon reasonable request to the corresponding author.
