# Supplementary material for: Pre-treatment tumour PET metrics and clinical outcomes of anal cancer in patients living with and without HIV
Source: Acta Oncol. 2025 Apr 24;64:40680. doi: 10.2340/1651-226X.2025.40680 (PMC12041796; doi:10.2340/1651-226X.2025.40680)

Supplementary material has been published as submitted. It has not been copyedited, or typeset by Acta Oncologica

Supplementary Figure 1. Example of the semiautomatic gradient-based segmentation-tool algorithm (PET_Edge) used to define the primary tumor and calculate primary-tumor PET metrics, including SUVmax, MTV, and TLG. Primary gross tumor volume was contoured within the anal canal. SUVmax was defined as the voxel with the highest quantified SUV avidity. TLG was defined as MTV multiplied by SUVmean of that volume.

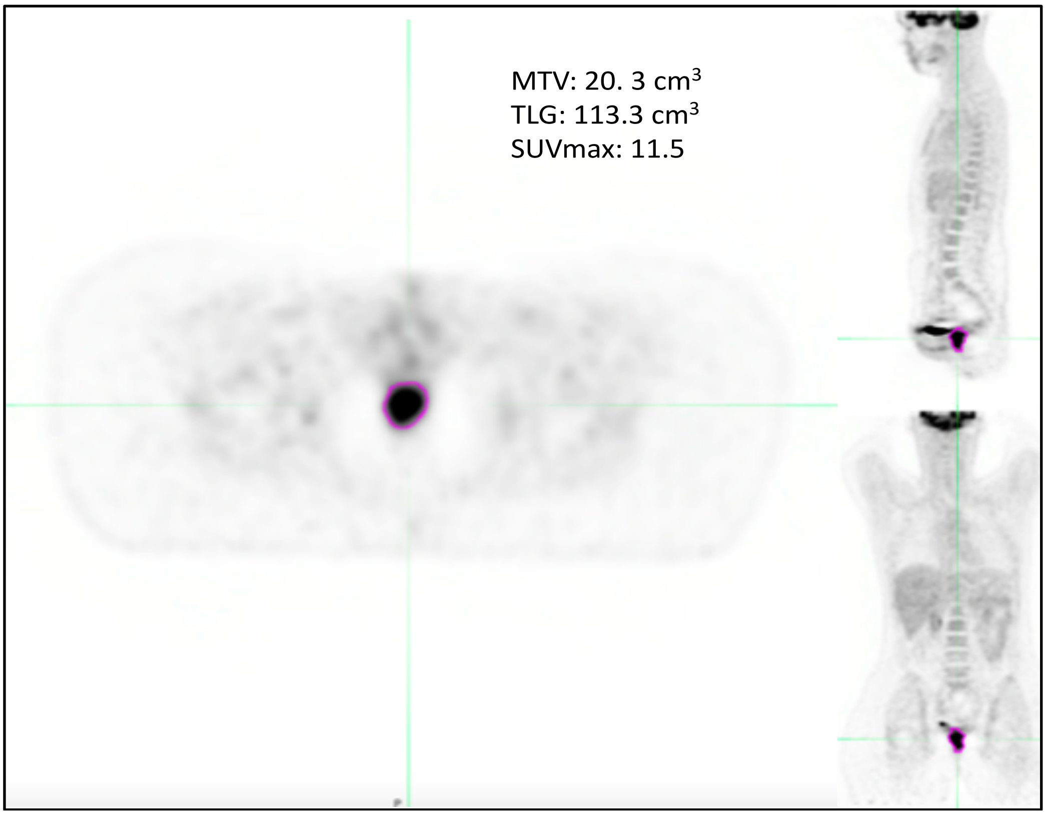

Supplement: Pre-treatment tumour PET metrics and clinical outcomes of anal cancer in patients living with and without HIV [file AO-64-40680-s1.pdf]
